# Temporal regularity of intrinsic cerebral activity in patients with chronic primary insomnia: a brain entropy study using resting‐state fMRI

**DOI:** 10.1002/brb3.529

**Published:** 2016-07-14

**Authors:** Fuqing Zhou, Suhua Huang, Lei Gao, Ying Zhuang, Shan Ding, Honghan Gong

**Affiliations:** ^1^ Department of Radiologythe First Affiliated HospitalNanchang UniversityNanchangChina; ^2^ Jiangxi Province Medical Imaging Research InstituteNanchangChina; ^3^ Department of RadiologyJiangxi Province Children's HospitalNanchangChina; ^4^ Department of OncologyThe Second Hospital of NanchangNanchangChina

**Keywords:** chronic primary insomnia, functional connectivity, functional plasticity, resting‐state functional MRI, sample entropy

## Abstract

**Introduction:**

Several neuroimaging studies have suggested that patients with chronic primary insomnia (CPI) exhibit anatomical and functional alterations of the brain, but the temporal regularity in spontaneous neuronal activity remains unknown. Here, brain entropy (BEN), a data‐driven method used to measure the signal regularity of a time series, was applied for the first time to investigate changes in the entire brain at the voxel level.

**Methods:**

Resting‐state functional MRI data were used to investigate insomnia‐related BEN alterations and the resting‐state functional connectivity (rsFC) pattern in seed regions with altered BEN in 29 patients with identified and untreated CPI and 29 matched healthy controls. Subsequently, within the CPI group, correlation analysis was conducted to evaluate the relationship between the clinical variables and the BEN and rsFC of the abnormal regions.

**Results:**

Chronic primary insomnia patients showed significant increase in BEN in the central part of the default‐mode network (DMN), the anterior regions of the task‐positive network (TPN), the hippocampus (Hipp), and basal ganglia (BG), and decreases in BEN in the right postcentral gyrus (PoCG) and right temporal–occipital junction (TOJ). We also demonstrated that three altered resting‐state functional connectivity (rsFC) patterns were associated with abnormal BEN regions in CPI patients. Correlation analysis identified an association between the altered rsFC and clinical variables, such as the Pittsburgh Sleep Quality Index (PSQI), in CPI patients.

**Conclusions:**

Together, these findings suggest that abnormal BEN‐related intrinsic functional plasticity in CPI patients corresponds to poor sleep quality during chronic insomnia. Alterations in both BEN and its affected connectivity may improve our understanding of treatment‐naïve CPI patients and promote the future development of new therapeutic strategies.

## 1 Introduction

Chronic primary insomnia (CPI) is defined as chronic difficulty in falling asleep or maintaining sleep or as nonrestorative sleep accompanied by significantly impaired daytime functioning in the absence of a specific physical, mental, or substance‐related cause (Benca, 2005). Insomnia affects 6%–20% of the general population and is associated with a variety of physical and psychiatric disorders (Benca, 2005). However, the underlying neural mechanisms remain largely unknown and have attracted much attention.

Recent structural neuroimaging studies have suggested the presence of anatomical alterations in primary insomnia, particularly in the hippocampus (Hipp) (Riemann et al., 2007), anterior cingulate cortex (Winkelman et al., 2013), and orbitofrontal cortex (OFC) (Altena, Vrenken, Van Der Werf, van den Heuvel, & Van Someren, 2010; Joo et al., 2013). Functional neuroimaging has also revealed abnormalities in cognitive and emotional processing in patients with primary insomnia (Drummond et al., 2013; Killgore, Schwab, Kipman, Deldonno, & Weber, 2013). For example, decreased functional connectivity between the amygdala and the insula, striatum, and thalamus suggests the existence of insomnia‐related dysfunction in the emotional circuit (Huang et al., 2012). In addition, functional magnetic resonance imaging (fMRI) has provided more evidence to support the hyperarousal theory (Riemann et al., 2010) in insomnia, which states that increased functional connectivity between the amygdala and the motor cortex (Huang et al., 2012) as well as the superior parietal lobe (SPL) and the premotor/supplementary motor cortex (SMA) demonstrates compensatory psychomotor performance (Huang et al., 2012; Li et al., 2014) and increased anterior insula coactivation with salience networks that are involved in the hyperarousal state (Chen, Chang, Glover, & Gotlib, 2014). In insomnia patients, cerebral functional reorganization or plasticity that is secondary to structural damage has been accepted as an important disease mechanism (Chee, 2013; O'Byrne, Berman Rosa, Gouin, & Dang‐Vu, 2014; Stoffers et al., 2014). However, the brain is a dynamic intrinsic connectivity network. It is a spatiotemporal continuum that involves both spatial and temporal patterns. The spatial patterns of intrinsic activity in patients with primary insomnia have been evaluated in terms of functional connectivity, as in the above‐mentioned resting‐state fMRI (rs‐fMRI) studies (Chen et al., 2014; Huang et al., 2012; Li et al., 2014; O'Byrne et al., 2014). However, it remains unclear whether the temporal patterns of neuronal activity are altered in CPI patients, and the distribution of intrinsic networks in the regions with altered temporal patterns is unknown. This knowledge may improve the understanding of the mechanisms of impaired neural function in this disorder.

Entropy is a statistical index that measures the activity irregularity of a dynamic system, and higher entropy indicates increased activity randomness in a system, making it less predictable. Brain entropy (BEN), a data‐driven voxel‐based sample entropy approach used to characterize brain temporal dynamics as a key feature, may provide a physiologically meaningful approach to assess the dynamic status of intrinsic activity in temporal patterns (Bassett, Nelson, Mueller, Camchong, & Lim, 2012; Bergstrom, 1969; Singer, 2009; Wang, Li, Childress, & Detre, 2014). The relatively lower BEN in the neocortex may reflect the “higher” mental functions performed by the cortex or more coherent neuronal population behavior (Wang et al., 2014). Neurophysiological electromagnetic studies argue that neural rhythms or regular activity could offer distinct and adapted computational solutions in various aspects of perception and cognition, such as long‐distance communication across brain regions, segmentation of sensory input, and memory (Arnal & Giraud, 2012; Rakic, 2009). BEN provides a relatively new method to explore the complexity of brain fMRI dynamics as well as their alterations in disease (Wang et al., 2014). BEN‐related estimators have been well established for studies of schizophrenia (Bassett et al., 2012; Sokunbi, Gradin et al., 2014; Sokunbi, Fung et al., 2014), attention deficit hyperactivity disorder (Sokunbi, Gradin et al., 2014; Sokunbi, Fung et al., 2014), drug abuse (Wang et al., 2013), multiple sclerosis (Zhou et al., 2016), and normal aging (Sokunbi et al., 2011; Yao et al., 2013).

**Figure 1:**
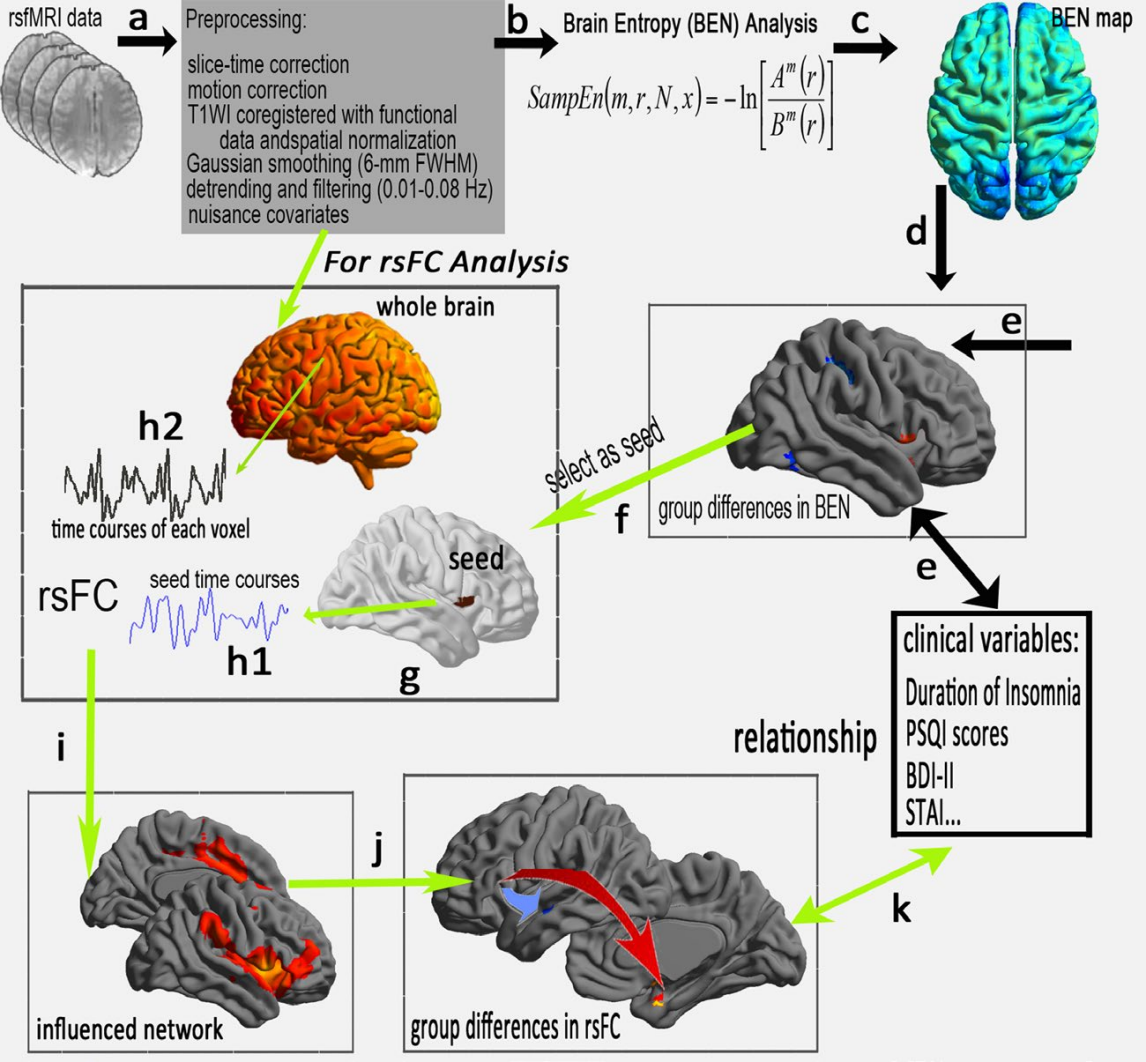
A flowchart of BEN and the affected connectivity pattern in CPI patients. (a) Preprocessing of rs‐fMRI data. (b) Sample entropy was then used to construct the BEN map in this study. (c) Visualization of the BEN map by group level. (d) Group comparison of BEN and (e) the clinical metrics associated with the altered BEN in CPI patients. To identify the specific networks influenced by BEN, (f) the region with altered BEN was selected as the region of interest (seed, g), and the time series was then extracted in the seed (h1) and each voxel of the whole brain (h2). (i) Calculated rsFC correlations and the constructed influenced network, and (j) a group comparison between the CPI and HC groups. Finally, statistical analysis of the relationship between the clinical metric and the rsFC values measured in the abnormal rsFC region between groups (k)

In this study, we aimed to explore insomnia‐related BEN alterations in CPI patients during the resting state. We hypothesized that the irregularity of the time series based on BEN observations could affect the relevant functional connectivity. To test the above hypothesis in CPI patients, we compared resting‐state functional connectivity (rsFC) in seed regions with altered BEN to those of healthy controls (HCs). In addition, within the CPI group, correlation analysis was conducted to evaluate the relationship between clinical variables and the BEN and rsFC values of abnormal regions (Fig. 1). Together, these results could reveal brain activity abnormalities in temporal patterns and spatial connectivity patterns based on temporal correlations in patients suffering from chronic insomnia.

## 2 Materials and Methods

The Medical Research Ethics Committee and the Institutional Review Board of the First Affiliated Hospital of Nanchang University approved the present study. This study was performed according to the approved guidelines and conducted in compliance with the principles of the Declaration of Helsinki. All subjects provided written informed consent for participation in this study.

### 2.1 Participants

The CPI patients and the HCs were recruited from the local hospital and the community, respectively, between May 2012 and July 2013. Each participant was assessed with a detailed clinical interview, a physical examination, and a clinical follow‐up. The inclusion criteria for the CPI group were: (1) 25–65 years of age; (2) an independent psychiatric syndrome (primary insomnia) as defined by the Diagnostic and Statistical Manual of Mental Disorders, version 4; and (3) duration of insomnia ≥1 year. All patients had reported having difficulty initiating or maintaining sleep or having nonrestorative sleep with resulting daytime distress or dysfunction that was not attributable to another medical or psychiatric disorder. Exclusion criteria for both the patients and HCs were: (1) clinical evidence of any moderate to severe sleep disorder other than insomnia (e.g., obstructive sleep apnea, restless legs syndrome); (2) abnormal sleep–wake rhythms; (3) hypertension, diabetes, or heart or respiratory diseases; (4) a history of cerebrovascular disease; (5) other neurological (neurodegenerative, epilepsy, head injury) or psychiatric (psychosis, current depression) diseases; (6) alcohol or illicit drug abuse or current intake of psychoactive medications; (7) a structural lesion identified on a brain MR image; or (8) MRI contraindications, such as claustrophobia or metallic implants or devices in the body. To ensure the quality of the rs‐fMRI data, each patient was limited to a maximum displacement in any cardinal directions (*x*,* y*,* z*) of <2 mm and a maximum rotation (*x*,* y*,* z*) of <2° (see the rs‐fMRI data preprocessing section). At the end of the scanning sessions, all participants reported that they had not fallen asleep during the scan. Finally, 29 treatment‐naïve CPI patients and 29 right‐handed gender‐, age‐, and education level‐matched HCs were enrolled. The participants were instructed to avoid caffeine, alcohol, central nervous system‐active agents, or any other psychoactive substances for 48 hr before the rs‐fMRI study. The clinical and demographic data of the 29 treatment‐naïve CPI patients and 29 right‐handed gender‐, age‐, and education level‐matched HCs are shown in Table 1.

**Table 1:** Clinical and demographic characteristics of CPI patients and HCs

Characteristic	CPI patientsMean (SD)	Healthy controlsMean (SD)	*p*‐values
Age, year	43.1 (11.4)	41.6 (11.5)	.625
Education, year	9.51 (2.93)	10.45 (4.03)	.319
Duration of insomnia, year	10.84 (8.58)	n/a (n/a)	n/a
STAI‐s	27.79 (4.23)	25.96 (7.93)	.278
STAI‐t	31.93 (4.79)	28.82 (9.83)	.132
BDI‐II	6.07 (5.14)	5.03 (1.64)	.306
PSQI	13.4 (2.37)	0.86 (1.05)	<.001
Mean head motion^a^	0.044 (0.024)	0.045 (0.041)	.965

BDI, Beck Depression Inventory; CPI, chronic primary insomnia; n/a, not applicable; PSQI, Pittsburgh Sleep Quality Index; SD, standard deviation; STAI‐s, the State Trait State‐Anxiety Inventory; STAI‐t, the State Trait Trait‐Anxiety Inventory. The *p* value was obtained by an independent sample two‐tailed *t*‐test.

a Head motion according to the criteria of Van Dijk. The same abbreviations apply for all figures and tables.

### 2.2 MRI data acquisition

Imaging was performed on a Trio 3.0 Tesla MRI system scanner (Siemens, Erlangen, Germany). Imaging data were acquired using the following sequences: (1) 240 rs‐fMRI images were acquired using a standard T2*‐weighted gradient echo sequence with the following parameters: repetition time/echo time = 2000/30 ms; field of view = 220 × 220 mm; matrix = 64 × 64; and 30 interleaved axial slices with 4‐mm thickness with an interslice gap of 1.2 mm. Subjects were instructed to keep their eyes closed, to avoid thinking about anything in particular, and to stay awake. (2) High‐resolution T1‐weighted anatomic images were obtained using a three‐dimensional magnetization prepared rapid acquisition gradient echo sequence with the following parameters: repetition time/echo time = 1900 ms/2.26 ms; matrix = 240 × 256; field of view = 215 mm × 230 mm; 176 sagittal slices with 1.0 mm slice thickness; and no gap. (3) Conventional T2‐weighted and fluid‐attenuated inversion recovery imaging protocols were used for diagnosis.

### 2.3 Rs‐fMRI data preprocessing

Functional image preprocessing was performed using Data Processing Assistant for Resting‐State fMRI, advanced edition, V2.3 (RRID: SCR_002372, http://www.restfmri.net) based on the Statistical Parametric Mapping software (SPM8, RRID: SCR_007037, http://www.fil.ion.ucl.ac.uk/spm/software/spm8/) running on Matlab 7.14.0 (Mathworks, Natick, MA, USA). This preprocessing was performed with standard processing steps (Yan, Craddock, Zuo, Zang, & Milham, 2013) as follows. The first 10 images from each subject were discarded during data acquisition to eliminate magnetic saturation effects, and the remaining 230 images were corrected for slice timing and realigned for intervolume head motion. Subjects were included if their head movement was <2 mm of translation along any axis and <2° of angular rotation along any axis during the rs‐fMRI scan. We also evaluated the group differences in head motion among CPI patients and HCs according to the criteria of Van Dijk, Sabuncu, & Buckner (2012) (Table 1). The high‐resolution individual T1‐weighted images were coregistered to the mean functional image after motion correction using a linear transformation, and the images were segmented into gray matter, white matter, and cerebrospinal fluid tissue maps as unified segmentation algorithm and a priori SPM tissue maps as reference. The coregistered T1‐weighted images were then resegmented using the custom tissue templates as reference images. All functional images were then resampled to 3‐mm cubic voxels and 6‐mm spatial smoothing with a full‐width half‐maximum Gaussian kernel, linear detrending, and temporal band‐pass filtering (0.01 Hz < *f* < 0.08 Hz) to eliminate high‐frequency noise and low‐frequency drift. Finally, the rs‐fMRI data were subjected to temporal correction of nuisance covariates, including head motion, white matter, and cerebrospinal fluid, but lacked global signals as the covariantes (Smith, Yan, & Wang, 2014).

### 2.4 BEN analysis

Brain entropy mapping was performed with the brain entropy mapping toolbox (RRID: SCR_014470, https://cfn.upenn.edu/~zewang/BENtbx.php) (Wang et al., 2014), which was calculated according to the sample entropy.

(1) $$\text{SampEn}\left(m,r,N,x\right)=-\text{ln}\left[\frac{A^{m}\;\left(r\right)}{B^{m}\;\left(r\right)}\right]$$

(2) $$B^{m}\;\left(r\right)=\frac{1}{\left(N-m)(N-m-1\right)}\sum_{i=1}^{N-m}\;B_{i}^{m}\;\left(r\right)$$

(3) $$A^{m}\;\left(r\right)=\frac{1}{\left(N-m)(N-m-1\right)}\sum_{i=1}^{N-m}\;B_{i}^{m+1}\;\left(r\right)$$

where *N* is the number of time points. Sample entropy starts by forming a series of embedded vectors that each has *m* consecutive points extracted from *x*:* u*_*i*_ = [*x*_*i*_, *x*_*i*_ = 1, ··· *x*_*i*_ + *m*−1], where *i *=* *1 to *N* − *m* + 1, and *m* is the predefined dimension; *r* is the prespecified distance threshold, and different *r* values (*r* = .3, .4, .5, .6, .7) were considered in this study; $B_{i}^{m}\left(r\right)$ counts the number of *u*_*j*_ (*j* = 1, to *N* − *m*, and *j* ≠ *i*) whose distances (Chebyshev distance is generally used, but any other distance can also be used) to *u*_*i*_ are less than *r,* such that $B_{i}^{m}+1\left(r\right)$ for the dimension of *m* + 1. More detailed information is available in the original publication (Wang et al., 2014).

Each individual BEN map was registered into a standard brain space, subject to 6‐mm full‐width half‐maximum smoothing and *z*‐transformed with Fisher's *r*‐to‐*z* transformation for group‐level analysis.

### 2.5 Statistical analysis for BEN data

A general linear model analysis was performed with the SPM8 toolkit to investigate group differences in BEN between CPI patients and HCs after controlling for the effects of age and gender. The significance level was thresholded at a family‐wise error (FWE) rate derived by using the Gaussian random field theory (voxel level |*z*| > 2.3 corresponding *p *<* *.0107 and cluster level *p *<* *.05). To determine whether BEN varied with disease progression, linear regression correlation analyses among the duration of insomnia, sleep quality index (PSQI), and the BEN of the obtained regions with significant group differences were then performed using the Statistical Package for the Social Sciences (SPSS) version 13.0 (Chicago, IL, USA) after controlling for gender and age. All analyses performed in SPSS had a statistical significance level of *p *<* *.05 and were corrected for multiple comparisons using Bonferroni correction.

### 2.6 Seed‐based rsFC analysis of altered BEN regions

In rs‐fMRI data preprocessing, after temporal band‐pass filtering, an additional nuisance linear regression was performed using white matter, cerebrospinal fluid, a whole‐brain signal, and six head motion parameters as covariates for a seed‐based rsFC analysis. The seed‐based rsFC analysis was conducted using Data Processing Assistant for Resting‐State fMRI, advanced edition, based on the seed regions with significant group differences in BEN. For each subject, the mean time course was extracted in each seed region, and Pearson's correlation coefficient between the mean time series of each seed region and that of each voxel in the entire brain was computed and converted to *z*‐values with Fisher's transformation to improve normality. Individual *z*‐values were then entered into a random‐effect, one‐sample *t*‐test in SPM8 to identify brain regions that showed significant positive correlations with each seed region for each group with a two‐tailed false discovery rate (FDR) corrected to *p *<* *.001. FDR‐controlling procedures provide less stringent control of Type I errors, and FDR could be used to construct the influenced network map for further group‐compare analyses. Therefore, this map was only thresholded using FDR. In this study, we only considered the brain areas with positive correlations with each seed as a mask for the group‐compare analysis because it has not been determined whether the anticorrelation is an artifact of the global signal regression in the rsFC analysis (Chai, Castañón, Öngür, & Whitfield‐Gabrieli, 2012; Murphy, Birn, Handwerker, Jones, & Bandettini, 2009).

### 2.7 Statistical analysis for rsFC data

Group differences in rsFC data between CPI patients and HCs were evaluated with a general linear model analysis in SPM8 after controlling for age and gender, and combining regions that exhibited significant positive connectivity. The threshold was a cluster‐level FWE‐corrected *p* value (*pFWE*, voxel level *p *<* *.0107 and cluster level *p *<* *.05) (see the group comparison of BEN). Finally, a linear regression correlation analysis was also performed in CPI patients to assess the relationship between abnormal clinical variables and the average rsFC measured in each region with altered rsFC in the two groups (*p *<* *.05 corrected for multiple comparisons using Bonferroni correction).

### 2.8 Statistical analysis for demographic and clinical variables

The differences in demographic and clinical data between the two groups were analyzed using an independent samples *t‐*test in SPSS 13.0. Differences were considered significant when *P* value was <.05.

## 3 Results

### 3.1 Clinical data and demographic profiling

The clinical and demographic characteristics of the study groups are shown in Table 1. Twenty‐nine CPI patients (19 females and 10 males) and 29 gender‐matched, right‐handed HCs with no visible abnormalities on conventional MRI were recruited. No significant differences in age (*t* = 0.491, *p *>* *.05) or years of education (*t* = −1.005, *p *>* *.05) were noted between CPI patients and HCs. As expected, the CPI patients exhibited significantly higher PSQI scores than did the HCs (*t* = 26.052, *p *<* *.001), while no significant differences were noted in the Beck Depression Inventory‐II (*t* = 1.033, *p *=* *.306), the State‐Trait‐State Anxiety Inventory (STAI‐s) (*t* = 1.095, *p *=* *.278), or the State‐Trait‐Trait Anxiety Inventory (STAI‐t) (*t* = 1.528, *p *=* *.132).

### 3.2 Comparison of differences in BEN between CPI patients and HC

We generated voxel‐wise BEN and further investigated the differences between the CPI and HC groups using rs‐fMRI data. The results in the between‐group differences were very similar, and they did not depend on the different prespecified distance thresholds (*r* = .3, .4, .5, .6, .7). Here, we report that the comparison of differences in BEN between CPI patients and HCs using the classical prespecified distance thresholds is *r* = .5. Other results from the comparison of differences in BEN using different prespecified distance thresholds are shown in Fig. S1.

**Figure 2:**
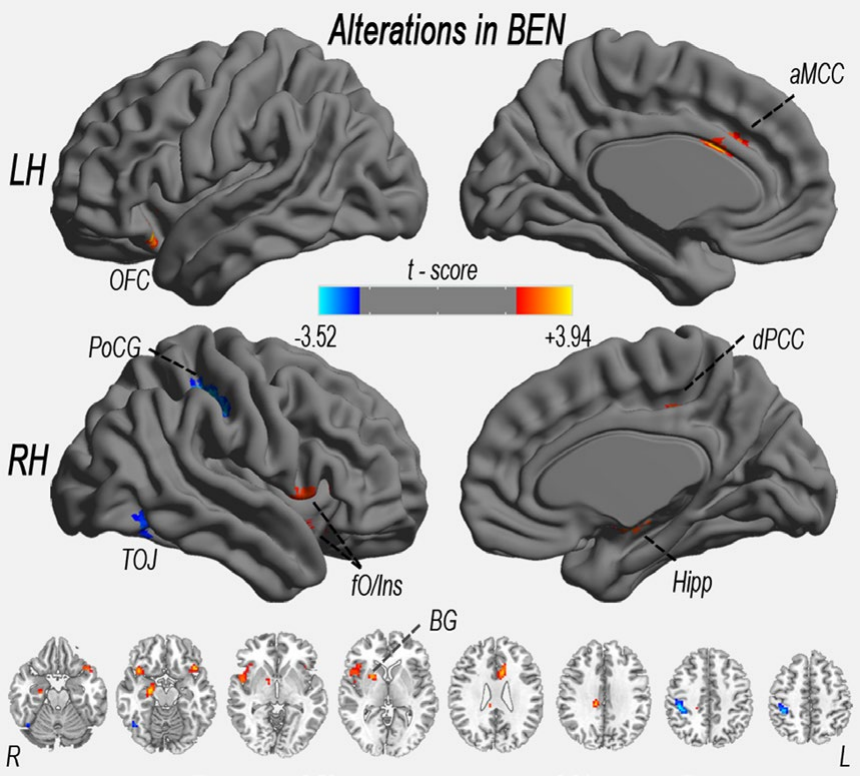
Significant increase (warm color) and decrease (cool color) in BEN in CPI patients (vs. healthy controls). The significance level was thresholded at a FWE rate derived using the Gaussian random field (GRF) theory (voxel level *p *<* *.0107 and cluster level *p* < .05). aMCC, anterior midcingulate cortex; BEN, brain entropy; BG, basal ganglia; CPI, chronic primary insomnia; Hipp, hippocampus; OFC, orbitofrontal cortex; fO/Ins, frontal operculum/insula; LH, left hemisphere; dPCC, dorsal posterior cingulate cortex; PoCG, postcentral gyrus; RH, right hemisphere; TOJ, temporal–occipital junction. The same abbreviations apply for all figures and tables

The BEN differences between the CPI patients and the HCs are shown in Fig. 2 and Table 2. Compared with the HCs, the CPI patients displayed increased resting BEN in the right dorsal posterior cingulate cortex (dPCC), the left anterior midcingulate cortex (aMCC), the left OFC, the right frontal operculum/insula (fO/Ins), the right Hipp, and the right basal ganglia (BG) (*pFWE* correction, voxel level *p *<* *.0107 and cluster level *p *<* *.05 are shown in Fig. 2). Significant decreases in resting BEN were observed in the right postcentral gyrus (PoCG) and right temporal–occipital junction (TOJ).

**Table 2:** Brain areas with significant differences in BEN between CPI patients and HCs (*pFWE* correction, voxel level *p *<* *.0107 and cluster level *p* < .05)

Cluster site	Peak MNI coordinates (*x*,* y*,* z*)	Peak intensity (*t*‐values)	Cluster size (voxel)
CPI patients > HC			
Right fO/Ins	39, 18, −12	3.59	154
Right dPCC	12, −30, 33	3.75	91
Right Hipp	24, −15, −18	3.66	58
Left aMCC	−6, 15, 24	3.48	57
Left OFC	−42, 18, −18	3.94	55
Right BG	21, 6, 3	3.33	39
CPI patients < HC			
Right PoCG	42, −33, 45	−3.52	109
Right TOJ	45, −60, −18	−2.88	30

MNI, Montreal Neurological Institute.

Moreover, in CPI patients, a linear regression analysis revealed no significant relationship between clinical measurements (including PSQI) and altered BEN (*p *>* *.05).

### 3.3 The rsFC differences in altered BEN regions between CPI patients and HCs

**Figure 3:**
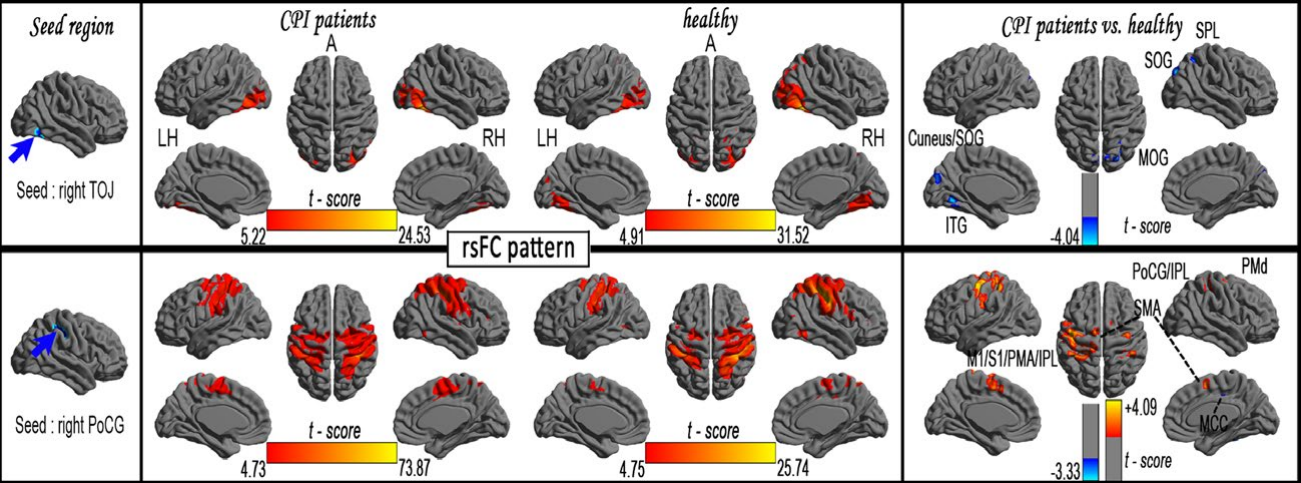
The rsFC patterns in each seed region with significantly reduced BEN in CPI patients (FDR corrected, *p *<* *.001) and rsFC differences between the CPI and HC groups

**Figure 4:**
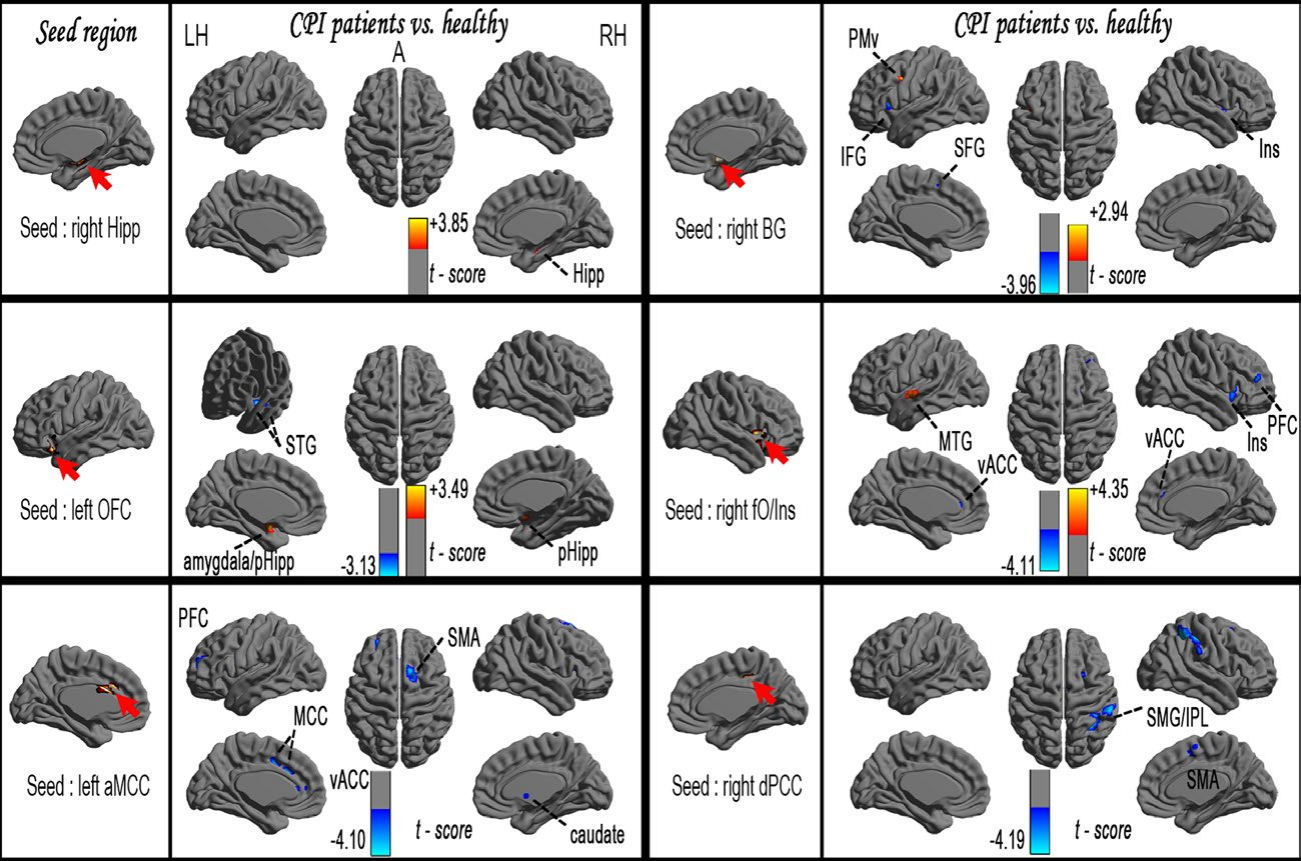
Significant increase (warm color) and decrease (cool color) in rsFC in CPI patients (vs. HCs), pFWE correction (voxel level *p *<* *.0107 and cluster level *p* < .05)

A one‐sample *t*‐test found that the rsFC patterns in all seed regions exhibited significant group differences in BEN between the CPI patients and the HCs (FDR corrected, *p *<* *.001) (Fig. S2 and Fig. 3). In the general linear model analysis, CPI patients exhibited the following characteristics relative to the HCs: (1) significantly decreased rsFC with the right TOJ, left aMCC, and right dPCC (*pFWE* correction, voxel level *p *<* *.0107 and cluster level *p *<* *.05) (Tables S1 and S2, Figs 3 and 4); (2) significantly increased rsFC with the right Hipp (*pFWE* correction, voxel level *p *<* *.0107 and cluster level *p *<* *.05); and (3) coexistence of significantly increased and decreased rsFCs with the right PoCG, right BG, left OFC, and right fO/Ins (*pFWE* correction, voxel level *p *<* *.0107 and cluster level *p *<* *.05).

### 3.4 Correlations between rsFC values and clinical variables in CPI patients

**Figure 5:**
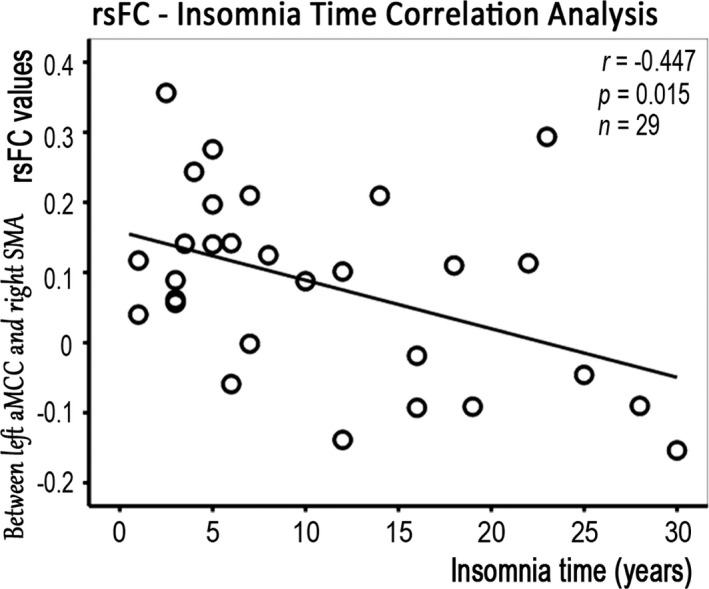
Relationship between the duration of insomnia and the rsFC values in CPI patients

**Figure 6:**
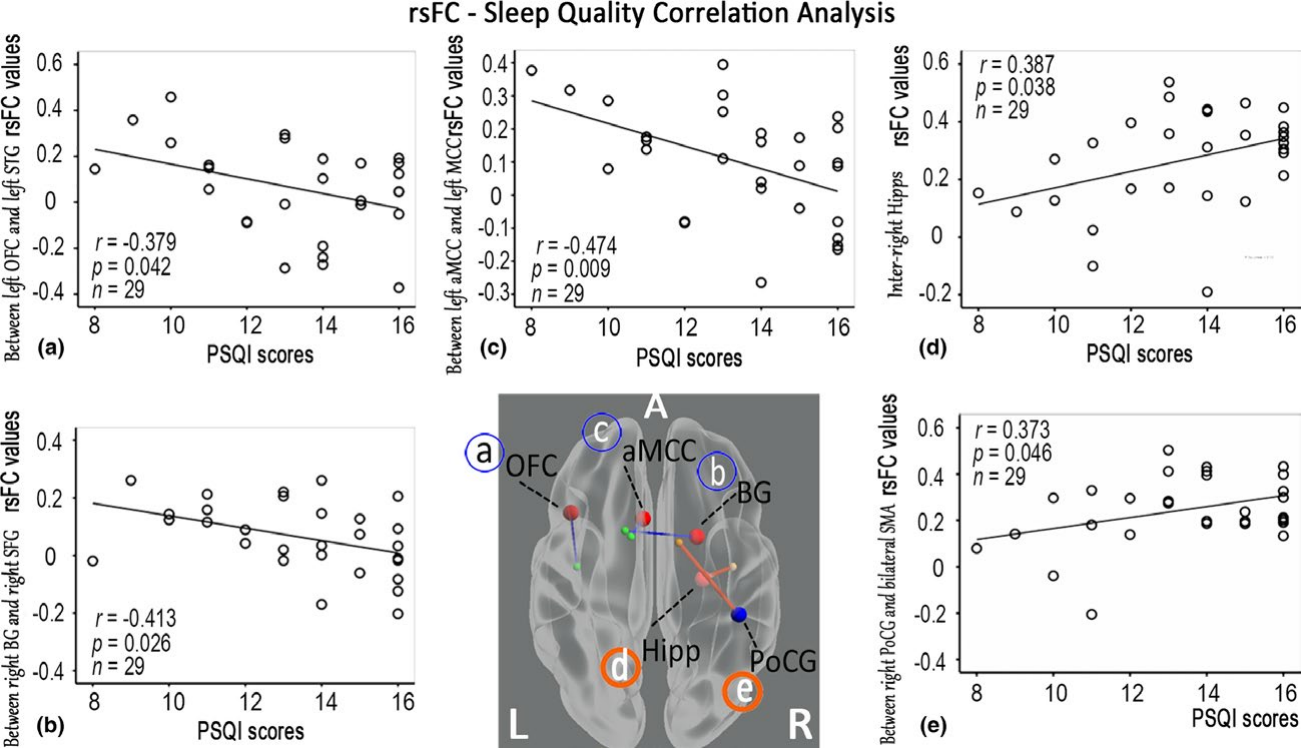
Relationship between the sleep quality indices and the rsFC values in CPI patients. PSQI scores were negatively correlated with reduced rsFC, but positively correlated with enhanced rsFC in CPI patients. The blue solid line signifies reduced rsFC areas connected with increased BEN (A, IFC; B, BG; C, aMCC), and the orange solid line signifies enhanced rsFC areas connected with altered BEN (D, Hipp; E, PoCG). The larger red sphere indicates increased BEN, the larger blue sphere indicates decreased BEN, and the smaller sphere indicates the rsFC connected areas

In CPI patients, a linear regression analysis revealed that the rsFC between the left aMCC and the right SMA was negatively correlated with the duration of insomnia (*p* < .05, corrected) (Fig. 5). In CPI patients, the sleep quality indices (PSQI scores) were negatively correlated with reduced rsFC between the left OFC and the left superior temporal gyrus, between the right BG and the right superior frontal gyrus (SFG), and between the left aMCC and the left MCC, but positively correlated with enhanced rsFC between the right PoCG and the bilateral SMA or with rsFC of the inter‐right Hipp (Fig. 6).

## 4 Discussion

To the best of our knowledge, this is the first study to investigate CPI‐related alterations in BEN and their affected connectivity patterns using rs‐fMRI. Three main findings were noted. (1) CPI specifically increased BEN in the right dPCC, left aMCC, left OFC, right fO/Ins, right Hipp, and right BG, but decreased BEN in the sensorimotor (right PoCG) and complex visual processing (right TOJ) regions. (2) We identified three rsFC patterns in altered BEN regions: (i) significantly decreased rsFC with the right TOJ, left aMCC, and right dPCC; (ii) significantly increased rsFC only with the right Hipp; and (iii) the coexistence of significantly increased and decreased rsFC with the right PoCG, right BG, left OFC, and right fO/Ins. (3) We also identified an association between the altered rsFC and clinical variables in CPI patients, such as the PSQI. This evidence contributes to understanding the neurobiological mechanisms that underlie insomnia.

### 4.1 Disturbances in the default‐mode network: increased BEN and disconnectivity

In this study, CPI patients demonstrated increased BEN in the right dPCC. This region belongs to the central part of the default‐mode network (DMN) and typically involves control of externally directed behavior. The DMN is an interconnected brain system that is most active when individuals are left to think to themselves undisturbed and is deactivated when individuals are directed toward task or goal (Andrews‐Hanna, Reidler, Huang, & Buckner, 2010; Andrews‐Hanna, Reidler, Sepulcre, Poulin, & Buckner, 2010). This network is involved in a wide range of higher order cognitive functions (Andrews‐Hanna, Reidler, Sepulcre et al. 2010). In a sense, CPI patients seem to exhibit a fluctuating disorder (Marques, Gomes, Clemente, dos Santos, & Castelo‐Branco, 2015). When CPI patients are awake during MRI, neuronal oscillation of the dPCC may contribute to both unrelated conscious mental activity and mental content. Increased BEN in the right dPCC may suggest that either irregular activity increases the dPCC's connectivity with both the DMN and the task‐positive network (TPN), which is involved in cognitive control in a wakeful state, or the right dPCC enables a lower level of information processing in the integration of the DMN with the TPN, which is associated with inefficient behavior.

In this study, we further observed a decrease in the rsFC to the right dPCC in the right supramarginal gyrus/inferior parietal lobule (IPL) and right SMA regions (Table S2). During insomnia, one interpretation of the hypoconnectivity of the right dPCC is that a lack of suppression primarily leads to abnormal cognitive activity. Increased entropy accompanying the disconnection of rsFC has been reported in the DMN in patients with attention deficit hyperactivity disorder (Sokunbi, Gradin et al., 2014; Sokunbi, Fung et al., 2014) and aging (Yao et al., 2013). It has also been reported that lower deactivation in the posterior cingulate cortex in insomniacs revealed a smaller increase in MR signals with increasing (N‐back working memory) task difficulty (Drummond et al., 2013). Sleep‐deprived participants also exhibited abnormal deactivation in the posterior cingulate cortex and decreased rsFC in the DMN during visual attention tasks (De Havas, Parimal, Soon, & Chee, 2012) and working memory tasks (De Havas et al., 2012; Gujar, Yoo, Hu, & Walker, 2010). In this study, the impairment of functional entropy of the right dPCC and the associated hypoconnectivity with certain regions of the TPN potentially serve as a candidate mechanism for the cognitive control deficits observed in CPI patients. We hope that future research can shed light on these hypotheses.

### 4.2 Disturbances in the TPN: increased BEN and distributed changes of rsFC

In this study, CPI patients also demonstrated increased BEN of the TPN in the left OFC, left aMCC, and right fO/Ins. The TPN comprises regions that are routinely activated during goal‐directed task performance, and these regions were previously called the executive control network and salience network (Fox et al., 2005). The OFC belongs to the executive control network and is associated with a wide range of sensory integration, emotion‐ and reward/punishment‐related cognitive processing function in decision making. A morphometry study revealed reduced gray matter volume in the left OFC that strongly correlated (*r *=* *.71) with the subjective severity of insomnia (Altena, Vrenken, Van Der Werf, van den Heuvel, & Van Someren, 2010). In addition Stoffers et al. (2012) noted that low gray matter density in the OFC area affects its role in sensing comfort. Comfort may be crucial for maintaining sleep in insomnia patients. The left aMCC and the right fO/Ins belong to the salience network. Recent studies have demonstrated that the aMCC, as part of the “dorsal” anterior cingulate cortex, is a hub that contributes to negative effects, pain, cognitive control, and ongoing behavioral adaption (Shackman et al., 2011; Sheth et al., 2012). In insomnia patients, an increase in the rostral anterior cingulate cortex volume and alterations in neurotransmission may relate to emotional dysregulation (O'Byrne et al., 2014). It has been suggested that the fO and insula are key nodes in the network for exerting control over cognitive processes. The fO and insula also play a critical role in maintaining sleep (Baglioni, Spiegelhalder, Lombardo, & Riemann, 2010), and increased insula coactivation within the salience network has been studied (O'Byrne et al., 2014). Functional neuroimaging has also revealed abnormalities in these areas in participants with insomnia or sleep deprivation (Chen et al., 2014; Joo et al., 2013); however, the findings have been inconsistent among neuroimaging studies (Riemann et al., 2007; Stoffers et al., 2014). In the present study, increased BEN in important nodes of the TPN indicates that the irregularity of neuronal dynamics is potentially associated with sensory integration and emotion‐related information processing. The above‐mentioned morphological and functional studies provided a foundation for understanding increased BEN in the TPN in this study.

With the exception of the hypoconnection of the left OFC and right fO/Ins, we observed hyperconnectivity in this study, which indicates functional compensation for abnormal intrinsic activity in the OFC or right fO/Ins (Tables S1 and S2, Figs 3 and 4). In addition, we observed that a longer duration of insomnia was associated with rsFC between the left aMCC and right SMA. The aMCC is an important region of SMA functional connections (Stevens, Hurley, & Taber, 2014), and these connection may be involved in coordinated unimanual motor behavior (Asemi, Ramaseshan, Burgess, Diwadkar, & Bressler, 2015; Shackman et al., 2011). It is therefore reasonable to speculate that chronic insomnia could skew the integration of synchronous activity in control during basic motor behavior.

### 4.3 Impairment in the Hipp and BG

The Hipp plays an important role in the consolidation of information from short‐term to long‐term memory and in spatial navigation. In this study, increased BEN and rsFC of the inter‐right Hipp indicated a regional impairment of the Hipp and internal compensation in CPI patients. Patients with primary insomnia demonstrated significantly reduced hippocampal volumes (Riemann et al., 2007), and a long duration of insomnia and poor quality sleep contribute to a decrease in the volume of the Hipp (Noh et al., 2012). Similarly, poor sleep quality is associated with cortical atrophy in the Hipp of community‐dwelling adults (Sexton, Storsve, Walhovd, Johansen‐Berg, & Fjell, 2014). Therefore, if hippocampal cortical atrophy also occurred in our patient population, the increased rsFC of the inter‐right Hipp correlates with poor sleep quality (as indicated by the PSQI) in patients with insomnia.

The BG is associated with a wide range of functions, including control of motor function, cognition, and emotion (Draganski et al., 2008). In this study, increased BEN also indicated functional impairment in the right BG. Further rsFC analysis between the right BG and right SFG in CPI patients has also suggested that sleep quality is associated with inter‐regional functional coordination due to the signal regularity of the BG in these patients.

### 4.4 Decreased BEN in the PoCG and TOJ

In this study, we identified decreased BEN (implying regular activity) in the right PoCG accompanied by hyperconnectivity in the sensorimotor network. Killgore et al. (2013) found that augmented functional connectivity may contribute to the sustained sensory processing of environmental stimuli and potentially prolong the latency to sleep. Our findings may reflect the hyper‐reactivity to a perceived threat in CPI patients that is consistent with the hyperarousal model in sustained sensory processing. Hyperactivity may prolong the latency to sleep in CPI patients (Killgore et al., 2013; Riemann et al., 2010).

In addition, a significant reduction in BEN was also noted in the left TOJ in the CPI group. In particular, the left TOJ exhibited reduced rsFCs compared with the right SPL, middle occipital gyrus, subesophageal ganglion (SOG), cuneus/SOG, and right cerebellum posterior lobe (CPL). The TOJs are joint areas with multiple functions, including the extraction of physical features of complex visual images (Nakamura et al., 2000). This reduced rsFC may reflect a general disruption of the complex visual information system in CPI patients and affect the modulation of visual tasks in individuals with insomnia.

### 4.5 The relationship between BEN and conventional spatial pattern analysis

Cerebral neuronal activity is a dynamic system comprising spatial components and time courses. However, the available analytical tools for rs‐fMRI data primarily examine spatial connectivity patterns. The other aspect of cerebral neuronal activity is temporal pattern analysis based on time courses, particularly signal regularity measures, and has largely been overlooked. In our study, the findings of a data‐driven BEN approach provide a solution to temporal patterns based on blood‐oxygenation‐level‐dependent time courses. Interestingly, most of the altered BEN regions in this study were associated with a wide range of processes. Therefore, BEN analysis expands and enriches the current understanding of CPI, which primarily stems from simple connectivity analyses that examine the functional alterations of spatial coherence.

Generally, the region with high complexity would be expected to show hypoconnectivity because it is harder to establish phase relationships, and the region with low complexity would show hyperconnectivity. In this study, however, we detected both hyper‐ and hypoconnectivity with the right PoCG, right BG, left OFC, and right fO/Ins, which may indicate cortical functional reorganization in CPI patients. The mechanism of functional reorganization remains unclear, but it represents an increased recruitment of psychomotor performance in the rsFC analysis (Huang et al., 2012; Li et al., 2014). BEN‐influenced intrinsic connectivity pattern analysis also indicated that the clinical association was with BEN‐influenced rsFC rather than with the BEN values themselves. One possible reason for this finding is that altered BEN regions are involved in emotional‐ or cognitive‐related multimodal information processing or lack corresponding sensitive indicators of clinical assessment. Taken together, a better understanding of how BEN affects functional connectivity and networks is needed. Future studies that combine temporal and spatial pattern analysis beyond simple replication could enable a closer examination that would bridge the gap in our understanding of the brain's resting state by measuring the four‐dimensional, blood‐oxygenation‐level‐dependent signal at rest. Fractional amplitude of low‐frequency fluctuation (fALFF) analysis, another improved temporal metric for detecting the regional intensity of spontaneous fluctuations in blood‐oxygenation‐level‐dependent signals as a reference context for BEN analysis, is reported in Appendix S1, while multiple time scale entropy (MSE), another entropy analysis of resting‐state fMRI that is a reliable metric of the temporal dynamic analysis of rs‐fMRI, is reported in Appendix S2.

## 5 Limitations

Several technical and biological limitations in the present study must be acknowledged. The first limitation of this analytical technique is the possible influence of excessive daytime sleepiness on resting‐state brain activity, which should be considered when interpreting our results. Second, we are aware that the sample size was too small to allow us to draw a definitive conclusion, and larger samples should be included in future studies. Finally, BEN mapping is still new in the literature, although it has exhibited promise in several clinical applications (Sokunbi, Gradin et al., 2014; Sokunbi, Fung et al., 2014; Wang et al., 2013, 2014). BEN calculations were based on sample entropy in this study, which has a prespecified distance threshold (*r* = .5 in this study) that potentially affected the estimated entropy but should not have affected the group‐level entropy contrast. This lack of effect was supported by additional analyses with various different sample entropy parameters (Fig. S1).

## 6 Conclusions

In this study, we observed that both BEN and its connectivity were altered in patients with CPI, indicating an abnormal BEN‐related intrinsic functional reorganization or plasticity. This knowledge may improve our understanding of the comprehensive neurobiological mechanisms in treatment‐naïve CPI patients and suggests that BEN is a useful measure to reveal changes in CPI's cerebral neuronal dynamics.

## Funding Information

This study was supported by the Science and Technology Support Program of Jiangxi, China (Grant/Award Number: “20151BBG70224”), the Science and Technology Project of Jiangxi Provincial Education Department (Grant/Award Number: “GJJ13136”), the National Science Foundation of China (Grant/Award Number: “81101041,” “81560284,” “81460263,” and “81260217”), the Natural Science Foundation of Jiangxi, China (Grant/Award Number: “2013BAB215008”).

## Conflict of Interest

The authors declare no competing financial interests and have not conflict of interest to declare.
