# Supplementary material for: Temporal regularity of intrinsic cerebral activity in patients with chronic primary insomnia: a brain entropy study using resting‐state fMRI
Source: Brain Behav. 2016 Jul 14;6(10):e00529. doi: 10.1002/brb3.529 (PMC5064341; doi:10.1002/brb3.529)
Supplement: Supplementary file 1 [file BRB3-6-e00529-s001.doc]

**Title: Temporal Regularity of Intrinsic Cerebral Activity in Patients with Chronic Primary Insomnia: A Brain Entropy Study Using Resting-state fMRI**

Fuqing Zhou ^1*,3^, Suhua Huang^2^, Lei Gao^1,3^, Ying Zhuang^4^, Shan Ding^2^, Honghan Gong ^1*,3^

^1^Department of Radiology, the First Affiliated Hospital, Nanchang University, Nanchang, Jiangxi Province, 330006, PRC; ^2^Department of Radiology, Jiangxi Province Children's Hospital, Nanchang, Jiangxi Province, 330006, PRC; ^3^Jiangxi Province Medical Imaging Research Institute, Nanchang, Jiangxi Province, 330006, PRC; ^4^Department of Oncology, The Second Hospital of Nanchang, Nanchang, Jiangxi Province, 330003, PRC.

**Appendix S1**

**Measurements of the fractional amplitude of low-frequency fluctuation in patients with chronic primary insomnia**

Spatial components and time courses are two important properties of intrinsic connectivity networks (ICNs)[^1^](#_ENREF_1). The spatial components of ICNs can be been evaluated by functional connectivity. The time courses of functional magnetic resonance imaging (fMRI) signals can be investigated by two categories of temporal patterns: signal complexity and low-frequency fluctuations [Wang et al., 2013; Wang et al., 2014]. Brain entropy (BEN) has been well established for studying the signal complexity of different brain states. The fractional amplitude of low-frequency fluctuation (fALFF), which is an improved approach for detecting the amplitude of low-frequency fluctuation (ALFF), has been proven to be a valuable characteristic of spontaneous neural activity [Zou et al., 2008]. In the present study, fALFF analysis was performed on chronic primary insomnia (CPI) patients and healthy controls (HCs) as a reference, and additional information is provided in the supplement report.

**Methods**

**fALFF Analysis**

Functional imaging preprocessing was performed using Data Processing Assistant for Resting-State fMRI, advanced edition, V2.3 ([http://www.restfmri.net](http://www.restfmri.net/)) based on the statistical parametric mapping software (SPM8, <http://www.fil.ion.ucl.ac.uk/spm/software/spm8/>) running on Matlab 7.14.0 (Mathworks, Natick, MA, USA). These preprocessing steps included: 1) dropping the first 10 images; 2) slice time correction; 3) three-dimensional motion correction; 4) linear detrending; 5) alignment with high-resolution individual T1-weighted images to the Montreal Neurological Institute (MNI) 152 template followed by resampling to 3-mm cubic voxels; and 6) spatial smoothing (full-width–half-maximum = 6 mm).

The fALFF qualifies the ratio of power of low-frequency fluctuations (0.01–0.08 Hz) to that of the entire frequency range and has been suggested to be more sensitive than the ALFF in detecting spontaneous brain activity [Zou et al., 2008]. The fALFF map was standardized by transforming each individual data points to z-scores for group voxel-wise comparisons.

A general linear model analysis was performed with the SPM8 toolkit to investigate the group differences in the fALFF between CPI patients and HCs (covariates: age and gender). The significance threshold was a cluster-level, family-wise error (FWE) corrected P value (*pFWE*, voxel level |z| > 2.3 and cluster level *P* < 0.05).

**Results**

**Alterations in the fALFF in CPI Patients**

The fALFF differences between CPI patients and HCs are presented in Figure S4 and Table S3. Compared with the HCs, the CPI patients displayed decreased fALFF in the left cerebellum posterior lobe (CPL), right cerebellum anterior lobe (CAL), right CPL, left basal ganglia (BG), and right BG (voxel-level FWE corrected *P values (pFWE)* < 0.05 and cluster extent k > 10 voxels). Significantly increased fALFF was observed in the bilateral rectus gyrus, right postcentral gyrus (PoCG) and left inferior parietal lobule (IPL) (*pFWE* < 0.05 and cluster extent k > 10 voxels).


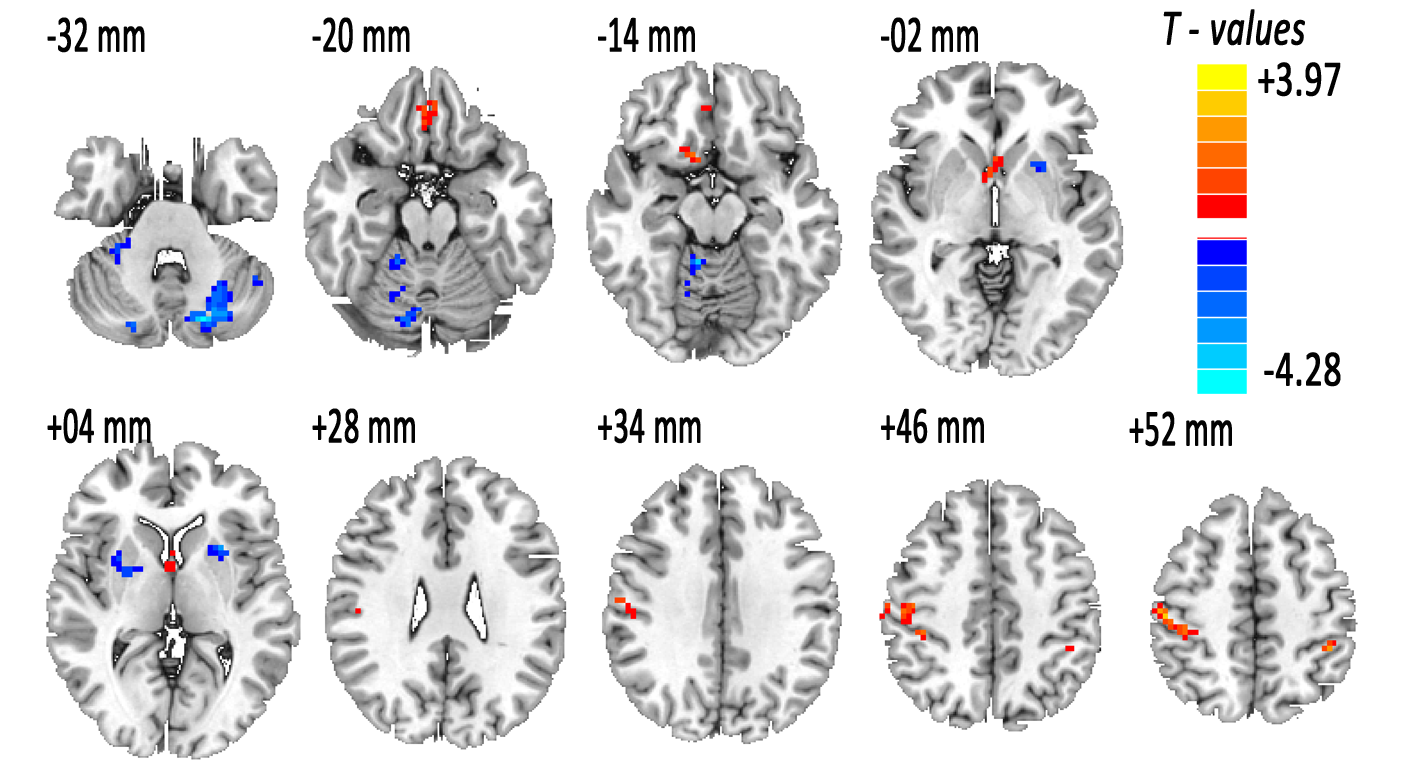


**Figure S4 Significant increase (warm color) and decrease (cool color) in the fALFF in CPI patients (vs. HCs), FWE correction, voxel level |z| > 2.3 and cluster level P < 0.05.**

***Table S3*** ***Brain areas with significant differences in the fALFF between CPI patients and HCs (pFWE******correction, voxel level |z| > 2.3 and cluster level P < 0.05)***

| Cluster site | Peak MNI  coordinates (x, y, z) | Peak intensity  (t-values) | Cluster size (voxel) |
| --- | --- | --- | --- |
| CPI patients < HC | | | |
| Left CPL | -24, -78, -30 | -4.28 | 182 |
| Right CAL | 9, -45, -15 | -3.68 | 72 |
| Right CPL | 21, -84, -27 | -3.33 | 50 |
| Left BG | -27, 12, 0 | -3.85 | 44 |
| Right BG | 30, 9, 6 | -3.38 | 42 |
| CPI patients > HCs | | | |
| Bilateral rectus gyrus | 0, 33, -24 | 3.51 | 34 |
| Right PoCG | 51,-24, 51 | 3.97 | 115 |
| Left IPL | -42, -42, 54 | 3.7 | 34 |

*Note: BG, basal ganglia; CAL, cerebellum anterior lobe; CPI, chronic primary insomnia; CPL,* *cerebellum posterior lobe; fALFF, fractional amplitude of low-frequency fluctuation; IPL, inferior parietal lobule; MNI, Montreal Neurological Institute; pFWE, family-wise error corrected P values; PoCG, postcentral gyrus.* *The same abbreviations apply for all figures and tables.*

**Discussion**

In this study, BEN and fALFF were used to detect alterations in signal complexity and low-frequency fluctuations in CPI patients, respectively. By comparing BEN to fALFF, we found that the fALFF alteration patterns in CPI only showed a minor overlap with the BEN alteration patterns in the right BG and right PoCG (Figure S5). This difference clearly indicates that BEN provides a different view of the temporal pattern that cannot be fully characterized by a certain frequency band of the entire signal. It is worth noting that the fALFF focuses on the intensity of low-frequency fluctuations.


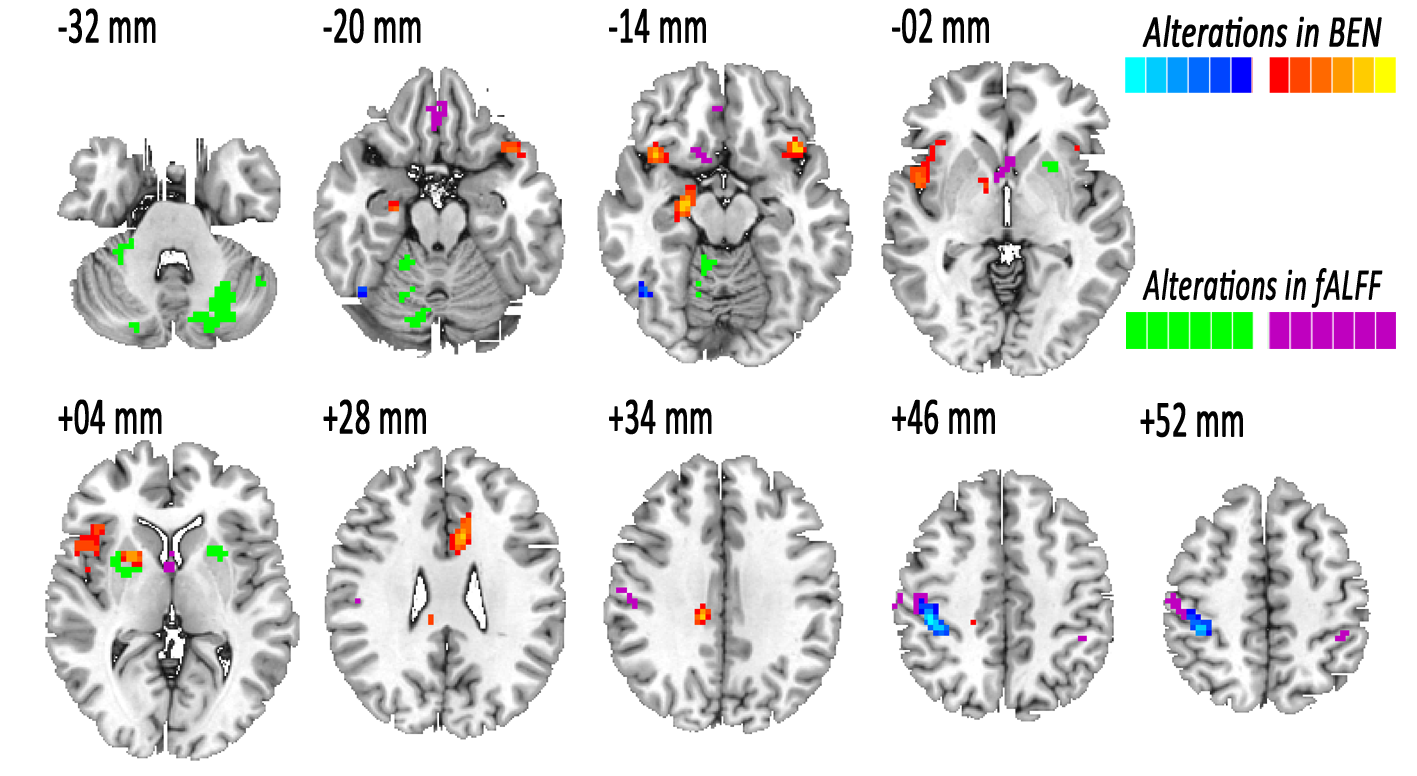


**Figure S5 Comparison of the differences between BEN and fALFF in CPI patients.**

**References**

Wang X, Jiao Y, Tang T, Wang H, Lu Z (2013): Investigating univariate temporal patterns for intrinsic connectivity networks based on complexity and low-frequency oscillation: a test-retest reliability study. *Neuroscience* **254:** 404-426.

Wang Z, Li Y, Childress AR, Detre JA (2014): Brain entropy mapping using fMRI. *Plos one* **9**: e89948.

Zou QH, Zhu CZ, Yang Y, Zuo XN, Long XY, Cao QJ, Wang YF, Zang YF (2008): An improved approach to detection of amplitude of low-frequency fluctuation (ALFF) for resting-state fMRI: fractional ALFF. *J Neurosci Methods* **172**: 137-141.
