# Supplementary material for: Temporal regularity of intrinsic cerebral activity in patients with chronic primary insomnia: a brain entropy study using resting‐state fMRI
Source: Brain Behav. 2016 Jul 14;6(10):e00529. doi: 10.1002/brb3.529 (PMC5064341; doi:10.1002/brb3.529)
Supplement: Supplementary file 2 [file BRB3-6-e00529-s002.docx]

**Title: Temporal Regularity of Intrinsic Cerebral Activity in Patients with Chronic Primary Insomnia:** **A Brain Entropy Study Using Resting-state fMRI**

Fuqing Zhou ^1*,3^, Suhua Huang^2^, Lei Gao^1,3^, Ying Zhuang^4^, Shan Ding^2^, Honghan Gong ^1*,3^

^1^Department of Radiology, the First Affiliated Hospital, Nanchang University, Nanchang, Jiangxi Province, 330006, PRC; ^2^Department of Radiology, Jiangxi Province Children's Hospital, Nanchang, Jiangxi Province, 330006, PRC; ^3^Jiangxi Province Medical Imaging Research Institute, Nanchang, Jiangxi Province, 330006, PRC; ^4^Department of Oncology, The Second Hospital of Nanchang, Nanchang, Jiangxi Province, 330003, PRC.

**Appendix S2**

**Measurements of multiple time scale entropy using resting-state functional magnetic resonance imaging in patients with chronic primary insomnia.**

Multiple time scale entropy (MSE) analysis has been well established for studying the complex dynamics of a time series and is a reliable metric of resting-state functional MRI (rs-fMRI) [Smith et al., 2014; Yang et al., 2015]. In this study, MSE analysis was performed on CPI patients and HCs as a reference, and additional information is provided in the supplement report.

**Methods**

**MSE analysis**

The rs-fMR images were preprocessed using Data Processing Assistant for Resting-State fMRI, advanced edition, V2.3 ([http://www.restfmri.net](http://www.restfmri.net/)) implemented in Matlab (MathWorks, Natick, MA). The re-fMRI data preprocessing included dropping the first 10 images, slice-timing correction, realigning, and normalizing to the standard space of the Montreal Neurological Institute (MNI) EPI template with high-resolution individual T1-weighted images that were resampled to a 3-mm cubic voxel. The covariates of the blood oxygenation level-dependent time series were regressed out before the MSE analysis was performed, including the time courses of six head motions and their derivatives and the cerebrospinal fluid. No global signal regression was performed to avoid introducing distortion into the time series data [Smith et al., 2014; Yang et al., 2015].

MSE calculation can be summarized in three steps: (a) constructing a coarse-grained time series according to different scale factors; (b) quantifying the sample entropy of each coarse-grained time series; and (c) examining the sample entropy profile over a range of scales. In this method, the length of each coarse-grained time series is equal to the length of the original time series divided by the scale factor. For Scale 1, the time series is the original time series.

Regional between-group differences in the whole-gray matter MSE mapping of each time scale were examined using the general linear model with age and gender as covariates. The significance threshold was a cluster-level FWE corrected P value (*pFWE*, voxel level |z| > 2.3 and cluster level *P* < 0.05).

**Results**

**Alterations of MSE in CPI Patients in Different Time Scales**

Figure S6 shows the regional between-group differences in the MSE of rs-fMRI signals among all scale factors at the voxel-wise level. Figure S7 shows the alterations of MSE in CPI patients in Scale 2.


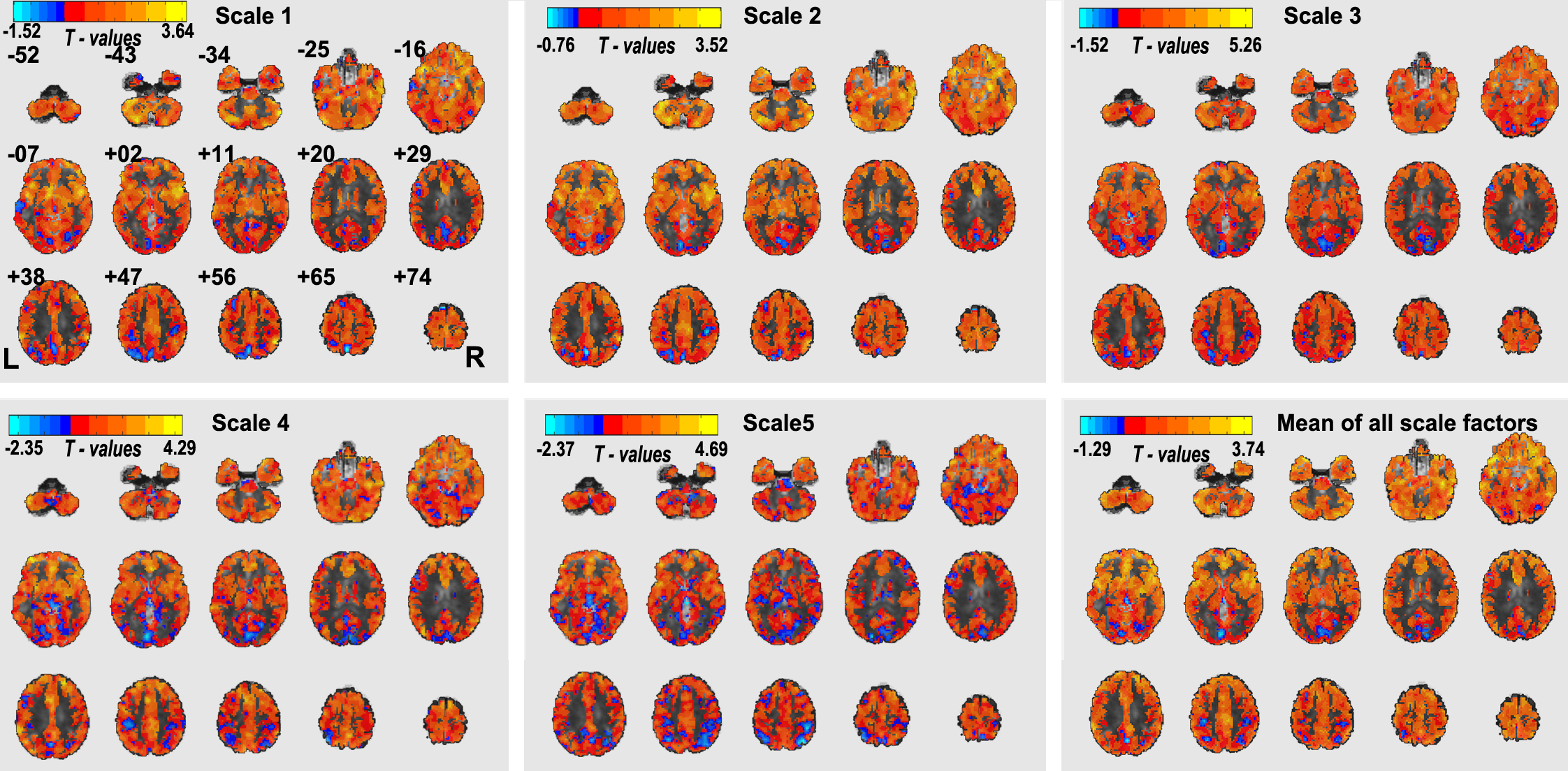


Figure S6. Regional differences in the MSE complexity of the rs-fMRI signal in all scale factors between CPI patients and HCs. A positive t value (warm color) represents a higher MSE value in CPI patients than in HCs, and the opposite is true with negative t values (cool color). A visual inspection suggested primarily patterns of pathologic change in CPI patients, indicating increased entropy (randomness) across all time scales (scales 1-5).


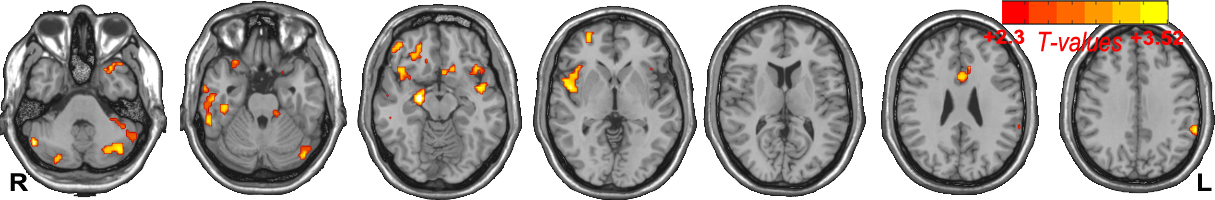


**Figure S7 Significant increased entropy in patients with CPI in Scale 2 (vs. HCs), FWE correction, voxel level P < 0.017 and cluster level P < 0.05.**

**Discussion**

In this study, there were similar regional differences in the MSE complexity and BEN analysis. The MSE method provides a profile of entropy across multiple time scales and, therefore, enables the extraction of a signature of complexity from physiologic signals that are not included in traditional methods based on the mean or variance or in conventional entropy methods used to measure entropy in a single time scale. The MSE profile enables the differentiation of true complexity from uncorrelated randomness, which does not contain meaningful information and can be regarded as a single-scale entropy method rather than a complex state, which is simply a measure of regularity.

**References**

Smith RX, Yan L, Wang DJ (2014): Multiple time scale complexity analysis of resting state FMRI. *Brain imaging and behav* **8**: 284-291.

Yang AC, Hong CJ, Liou YJ, Huang KL, Huang CC, Liu ME, Lo MT, Huang NE, Peng CK, Lin CP, Tsai SJ (2015): Decreased resting-state brain activity complexity in schizophrenia characterized by both increased regularity and randomness. *Hum Brain Mapp* **36**: 2174-2186.
