# Supplementary material for: Temporal regularity of intrinsic cerebral activity in patients with chronic primary insomnia: a brain entropy study using resting‐state fMRI
Source: Brain Behav. 2016 Jul 14;6(10):e00529. doi: 10.1002/brb3.529 (PMC5064341; doi:10.1002/brb3.529)
Supplement: Supplementary file 3 [file BRB3-6-e00529-s003.doc]

Title: Temporal Regularity of Intrinsic Cerebral Activity in Patients with Chronic Primary Insomnia: A Brain Entropy Study Using Resting-state fMRI

Fuqing Zhou ^1*,3^, Suhua Huang^2^, Lei Gao^1,3^, Ying Zhuang^4^, Shan Ding^2^, Honghan Gong ^1*,3^

^1^Department of Radiology, the First Affiliated Hospital, Nanchang University, Nanchang, Jiangxi Province, 330006, PRC; ^2^Department of Radiology, Jiangxi Province Children's Hospital, Nanchang, Jiangxi Province, 330006, PRC; ^3^Jiangxi Province Medical Imaging Research Institute, Nanchang, Jiangxi Province, 330006, PRC; ^4^Department of Oncology, The Second Hospital of Nanchang, Nanchang, Jiangxi Province, 330003, PRC.

Additional information:


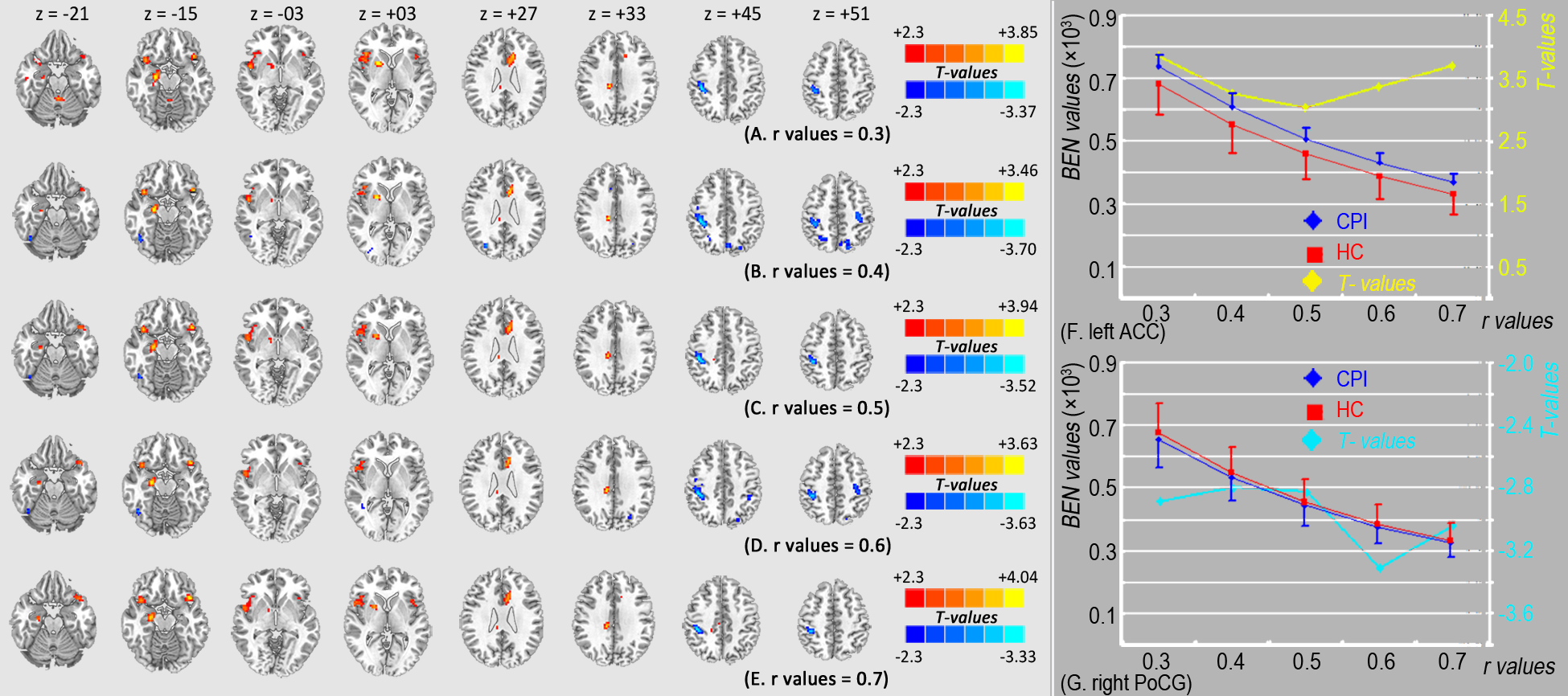


**Figure S1.** Voxel-wise BEN alterations in CPI patients (vs. HCs) in different pre-specified distance thresholds (r-values) (A-E), *pFWE* correction, voxel level *P* < 0. 017 and cluster level P < 0.05.

*Red and blue colors denote increased and decreased* *BEN, respectively. The color bars indicate the t-values. Images are displayed in radiological conventions. The altered pattern of the BEN values of the left aMCC (F) and the right PoCG (G) in different pre-specified distance thresholds (r-value). The broken (yellow and cyan) line signifies the compared t-values of two groups in different pre-specified distance thresholds (r-values). For example, the masks of the left ACC and the right PoCG were extracted based on the union of the altered BEN maps from the different pre-specified distance threshold conditions (aMCC, anterior midcingulate cortex; BEN, brain entropy; CPI, chronic primary insomnia; HC, healthy controls; PoCG, postcentral gyrus).*


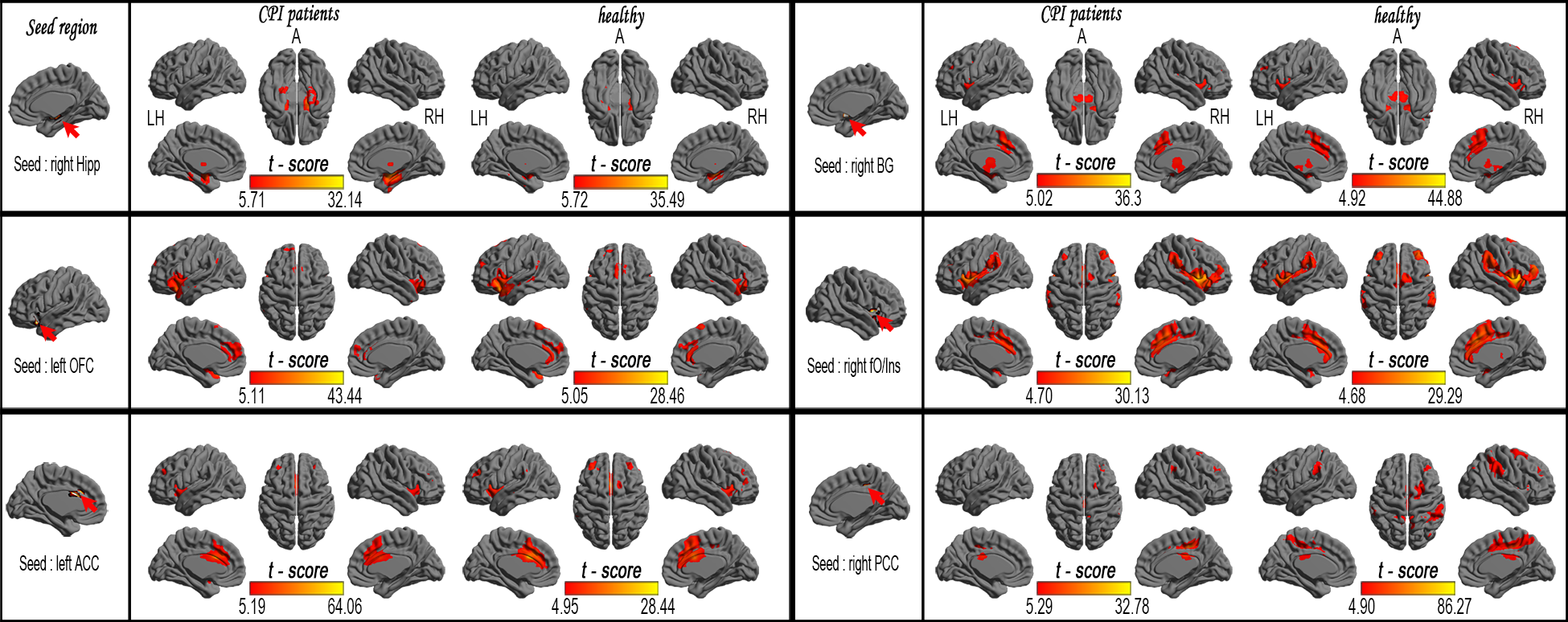


**Figure S2.** The rsFC patterns in each seed region with significantly enhanced BEN in CPI patients (FDR corrected, P < 0.001) visualized with BrainNet Viewer.

*ACC, anterior cingulate cortex; BEN, brain entropy; BG, basal ganglia; CPI, chronic primary insomnia; Hipp, hippocampus; IFC, inferior frontal cortex; Ins, insula; PCC, posterior cingulate cortex; PoCG, postcentral gyrus; rsFC, resting-state functional connectivity; TOJ, temporal occipital junction.*

**Table S1** Significant rsFC differences in seed regions with decreased BEN (CPI patients > HC)

| Brain regions | BA | Peak T-scores | MNI coordinates | | | Cluster size (voxels) |
| --- | --- | --- | --- | --- | --- | --- |
|  |  |  | x | y | z |  |
| **Seed: right TOJ** | | | | | | |
| Left CPL |  | -3.51 | -12 | -63 | -9 | 75 |
| Right MOG |  | -3.13 | 30 | -72 | 21 | 20 |
| Left cuneus/SOG | 19 | -4.04 | -12 | -78 | 24 | 45 |
| Right SOG | 19 | -3.15 | 24 | -81 | 36 | 51 |
| Right SPL | 7 | -2.87 | 24 | -60 | 54 | 14 |
| **Seed: right PoCG** | | | | | | |
| Right MCC |  | -2.94 | 18 | -24 | 42 | 17 |
| Left M1/S1/PMA/IPL | 3,4,6,40 | 4.08 | -48 | -3 | 54 | 490 |
| Right PoCG/IPL | 40 | 3.29 | 45 | -39 | 51 | 35 |
| Bilateral SMA | 6 | 3.79 | 12 | 3 | 60 | 84 |
| Right PMd | 6 | 2.93 | 33 | -9 | 60 | 16 |

*Note: CPL = cerebellum posterior lobe; PMd, dorsal premotor*

**Table S2** Significant rsFC differences in seed regions with increased BEN (CPI patients > HC)

| Brain regions | BA | Peak T-scores | MNI coordinates | | | Cluster size (voxels) |
| --- | --- | --- | --- | --- | --- | --- |
|  |  |  | x | y | z |  |
| Seed: right Hipp | | | | | | |
| Right Hipp | 20 | 3.84 | 39 | -9 | -15 | 27 |
| Seed: left OFC | | | | | | |
| Right pHipp | 28 | 3.39 | 21 | 3 | -24 | 18 |
| Left amygdala/pHipp | 34 | 3.48 | -24 | 3 | -18 | 27 |
| Left STG | 21 | -3.13 | -39 | -9 | -12 | 10 |
| Seed: right fO/Ins | | | | | | |
| Right Ins | 32 | -3.66 | 30 | 15 | 3 | 32 |
| Right PFC |  | -4.10 | 27 | 33 | 9 | 60 |
| Left vACC |  | -3.22 | -9 | 30 | 3 | 13 |
| Right vACC | 32 | -3.45 | 15 | -3 | 51 | 44 |
| Left MTG | 22 | 4.22 | -63 | -6 | -6 | 22 |
| Seed: right BG | | | | | | |
| Right Ins | 13 | -3.05 | 36 | 6 | 3 | 28 |
| Left IFG | 13 | -3.96 | -36 | 27 | 9 | 52 |
| Left SFG | 24 | -3.63 | -15 | 9 | 51 | 14 |
| Left PMv | 6 | 2.94 | -27 | 6 | 42 | 14 |
| Seed: right dPCC | | | | | | |
| Right SMG/IPL | 40 | -4.19 | 33 | -48 | 51 | 211 |
| Right SMA | 6 | -3.59 | 15 | 6 | 54 | 65 |
| Seed: left aMCC | | | | | | |
| Right caudate |  | -3.64 | 6 | 6 | 0 | 10 |
| Left IFG | 45 | -3.47 | -42 | 27 | 3 | 23 |
| Left PFC | 10 | -3.15 | -18 | 45 | 9 | 43 |
| Left vACC | 32 | -4.09 | -15 | 21 | 30 | 28 |
| Left MCC | 32 | -3.16 | -12 | 6 | 42 | 22 |
| Right SMA | 6 | -3.83 | 15 | 15 | 69 | 54 |

*Note: aMCC, anterior midcingulate cortex; fO, frontal operculum; IPL, inferior parietal lobule; IFG, inferior frontal gyrus; MCC, middle cingulate cortex; MTG,* *middle temporal gyrus; pHipp, parahippocampal pyrus; PMv, ventral premotor; PFC, prefrontal cortex; SFG, superior frontal gyrus; SMG,* *supramarginal gyrus; STG, superior temporal gyrus; vACC, ventral anterior cingulate cortex.*
